# Stimulation of the atypical chemokine receptor 3 (ACKR3) by a small-molecule agonist attenuates fibrosis in a preclinical liver but not lung injury model

**DOI:** 10.1007/s00018-022-04317-y

**Published:** 2022-05-13

**Authors:** Tom Van Loy, Steven De Jonghe, Karolien Castermans, Wouter Dheedene, Reinout Stoop, Lars Verschuren, Matthias Versele, Patrick Chaltin, Aernout Luttun, Dominique Schols

**Affiliations:** 1 Laboratory of Virology and Chemotherapy, Rega Institute, Department of Microbiology, Immunology and Transplantation, KU Leuven, Herestraat 49, 3000 Leuven, Belgium; 2 CISTIM, Gaston Geenslaan 2, 3001 Leuven, Belgium; 3 Endothelial Cell Biology Unit, Center for Molecular and Vascular Biology, Department of Cardiovascular Sciences, KU Leuven, 3000 Leuven, Belgium; 4 Department of Metabolic Health Research, The Netherlands Organisation for Applied Scientific Research (TNO), Leiden, The Netherlands; 5 Centre for Drug Design and Discovery (CD3), KU Leuven, Gaston Geenslaan 2, 3001 Leuven, Belgium

**Keywords:** Atypical chemokine receptor 3, Small molecule agonist, Lung, Liver, Fibrosis, Preclinical models

## Abstract

Atypical chemokine receptor 3 (ACKR3, formerly CXC chemokine receptor 7) is a G protein-coupled receptor that recruits β-arrestins, but is devoid of functional G protein signaling after receptor stimulation. In preclinical models of liver and lung fibrosis, ACKR3 was previously shown to be upregulated after acute injury in liver sinusoidal and pulmonary capillary endothelial cells, respectively. This upregulation was linked with a pro-regenerative and anti-fibrotic role for ACKR3. A recently described ACKR3-targeting small molecule agonist protected mice from isoproterenol-induced cardiac fibrosis. Here, we aimed to evaluate its protective role in preclinical models of liver and lung fibrosis. After confirming its in vitro pharmacological activity (i.e., ACKR3-mediated β-arrestin recruitment and receptor binding), in vivo administration of this ACKR3 agonist led to increased mouse CXCL12 plasma levels, indicating in vivo interaction of the agonist with ACKR3. Whereas twice daily in vivo administration of the ACKR3 agonist lacked inhibitory effect on bleomycin-induced lung fibrosis, it had a modest, but significant anti-fibrotic effect in the carbon tetrachloride (CCl_4_)-induced liver fibrosis model. In the latter model, ACKR3 stimulation affected the expression of several fibrosis-related genes and led to reduced collagen content as determined by picro-sirius red staining and hydroxyproline quantification. These data confirm that ACKR3 agonism, at least to some extent, attenuates fibrosis, although this effect is rather modest and heterogeneous across various tissue types. Stimulating ACKR3 alone without intervening in other signaling pathways involved in the multicellular crosstalk leading to fibrosis will, therefore, most likely not be sufficient to deliver a satisfactory clinical outcome.

**Supplementary Information:**

The online version contains supplementary material available at 10.1007/s00018-022-04317-y.

## Introduction

Atypical chemokine receptor 3 (ACKR3), formerly known as CXC chemokine receptor 7 (CXCR7), is a cell-surface receptor that belongs to the superfamily of G protein-coupled receptors (GPCRs). Chemokine receptors comprise ~ 20 GPCRs that are activated by a family of ~ 50 small secreted proteins, termed chemokines, which are best-known for mediating the recruitment and activation of different types of leukocytes. They also play important roles during development and contribute to tissue remodeling after injury [1, 2]. Many chemokines interact with multiple chemokine receptors and most receptors engage with multiple chemokines. Although this suggests the existence of signaling redundancy, it is becoming clear that different chemokines stimulate a particular chemokine receptor in different ways, reflecting their biased signaling properties [3]. ACKR3 interacts with two chemokine ligands: interferon-inducible T cell α chemoattractant [I-TAC, CXC chemokine ligand 11 (CXCL11)] and stromal cell-derived factor 1α [SDF-1α, CXC chemokine ligand 12 (CXCL12)] [4, 5]. CXCL12 also activates CXC chemokine receptor 4 (CXCR4), for which it is the sole ligand, while CXCL11 is also interacting with chemokine receptor CXCR3.

Upon ligand binding, the majority of chemokine receptors evoke intracellular signaling via the activation of heterotrimeric G proteins consisting of Gα and Gβγ subunits. Receptor activation enables the exchange of GDP for GTP at the Gα subunit, which causes dissociation (or rearrangement) of the Gα-GTP and Gβγ subunits, both of which further induce downstream signaling pathways. Phosphorylation of the receptor’s intracellular C-terminal tail leads to recruitment of β-arrestins, which are multifunctional proteins not only involved in receptor desensitization (i.e., shutting off G protein signaling) and internalization, but they also function as scaffold proteins that finetune additional downstream signaling pathways [6].

ACKR3 belongs to the atypical chemokine receptors for which four members are described (ACKR1-4) [7]. Unlike the classical GPCRs, ACKRs are devoid of functional G protein coupling, but retain the ability to recruit β-arrestins [8]. ACKRs are preferentially expressed on endothelial and epithelial cells at barrier sites and generally rather poorly on cells of hematopoietic origin [8, 9]. ACKR3 is thought to function as a decoy or scavenging receptor that shapes chemokine gradients by trapping CXCL12 and thus regulating its availability in the microenvironment and circulation [9]. ACKR3 also modulates the activity of CXCR4 when both receptors are co-expressed as heterodimers, at least in vitro [10, 11]. β-arrestin-dependent signaling downstream of ACKR3, including phosphorylation of extracellular regulated protein kinases (Erk1/2) and Akt, was also reported [12–15]. The underlying mechanism(s) of G protein-independent β-arrestin signaling is, however, still a matter of debate [16].

Liver sinusoidal endothelial cells (LSECs) are key players in the multicellular crosstalk that balances regeneration and fibrosis after liver injury [17]. Also in the injured lung the vascular niche [i.e., pulmonary capillary endothelial cells (PCECs)] regulates the balance between tissue regeneration and maladaptive healing [18]. Upon acute injury, ACKR3 expression is upregulated in mouse LSECs, which leads to increased expression of the transcription factor Id-1 and the concomitant release of paracrine growth regulators (i.e., angiocrine factors) that favor liver regeneration [17]. In cultured human LSECs a peptide-based ACKR3 agonist (TC14012), which also acts as a very potent CXCR4 antagonist [19, 20], induced Id-1 upregulation [17]. During chronic liver injury the early pro-regenerative effect of ACKR3-activity becomes overruled by increased CXCR4 expression and activity, which ultimately drives a pro-fibrotic response [17]. ACKR3 is also significantly upregulated in mouse PCECs after single intratracheal bleomycin administration. Furthermore, ACKR3 activity in PCECs protected against lung epithelial damage and ameliorated fibrosis by counteracting overactivation of Wnt signaling in PCECs under pathological conditions [18]. Recently, in vivo activation of ACKR3 by a potent and drug-like small molecule agonist was shown to decrease cardiac fibrosis in an isoproterenol-induced model of cardiac injury. In this model, the ACKR3 agonist blocked the CXCL12-scavenging function of ACKR3, which led to increased CXCL12 plasma levels. It was further suggested that cardiac function improved due to enhanced CXCR4 activity [21].

In this study we further embarked on the potential anti-fibrotic role of ACKR3 agonism by studying the effect of this previously described ACKR3-targeting small molecule agonist [21] in preclinical mouse models of lung and liver fibrosis. The ACKR3 agonist was resynthesized and its in vitro pharmacological properties were studied to confirm receptor potency, efficacy and specificity. The selective profile of this small molecule (i.e., being a potent ACKR3-agonist without CXCR4 antagonism) allowed us to evaluate the role of pure ACKR3-agonism, thus ruling out the potential additional effect of CXCR4 antagonism, in mouse models of lung and liver fibrosis.

## Materials and methods

### Compounds and proteins

The ACKR3 agonist (compound **18** from [21]) was custom synthesized by Aragen Life Sciences Pvt. Ltd. (India) according to the previously described procedure [21], commissioned by the Centre for Drug Design and Discovery (CD3). The ALK5/ALK4 kinase inhibitor SM 16 [22] was purchased from ChemScene (CS-0042167). Recombinant human CXCL12 and CCL5 were purchased from Peprotech (United Kingdom, UK). Human AlexaFluor647-labeled CXCL12 (CXCL12^AF647^) was obtained from Almac (UK).

### β-arrestin2 recruitment assay

β-arrestin2 recruitment downstream of ACKR3 activation was analyzed using the PathHunter CHO-K1 CXCR7 β-arrestin Cell Line (93-0248C2; DiscoverX). Cells were cultured in AssayComplete™ Cell Culture Kit-107 (92-3107G; DiscoverX) at 37 °C and 5% CO_2_. Briefly, 20,000 cells/well were seeded in AssayComplete™ Cell Plating 2 Reagent (93-0563R2A; DiscoverX) in a white 96-well plate (100 µL/well) with clear bottom (Costar, 3610) and incubated overnight at 37 °C and 5% CO_2_. The next day, 10 µL/well of test compound (11 × concentrated) was added and cells were incubated for 90 min at 37 °C and 5% CO_2_. Compounds were tested in duplicate in a 1:4 dilution series ranging from 1000 nM to 0.06 nM final concentration. After incubation, 55 µL/well of detection reagent (PathHunter Detection kit; 93-0001; DiscoverX) was added and cells were incubated for 1 h at room temperature (RT) protected from light. Finally, luminescence was measured using a FLIPR Tetra^®^ device (Molecular Devices, Sunnyvale, CA, USA). Four parameter non-linear curve fitting (GraphPad Prism 9.0.2) was used to determine the EC_50_ value for β-arrestin2 recruitment.

### CXCL12^AF647^ competition binding

Binding affinity to human (h)CXCR4 and human (h)ACKR3 was investigated with a competition binding assay using CXCL12^AF647^ (Almac, UK) as the tracer molecule [23]. Jurkat cells (human T lymphocytic cell line, hCXCR4 positive) or U87.CD4.ACKR3 cells (human glioblastoma cell line, hACKR3 positive) were resuspended in assay buffer [Hank’s Balanced Salt Solution (HBSS, Thermo Fisher Scientific), 20 mM HEPES buffer, 0.2% bovine serum albumin (Sigma-Aldrich), pH 7.4] at 3 × 10^5^ cells per sample and treated with increasing concentrations of compound at RT for 15 min. A fixed amount of CXCL12^AF647^ (2.9 nM and 1.45 nM in case of hCXCR4 and hACKR3, respectively) was added and cells were incubated at RT for 30 min protected from light. Cells were finally fixed in 1% paraformaldehyde in DPBS and the CXCL12^AF647^ binding signal [i.e., mean fluorescence intensity (MFI)] was quantified by flow cytometry (FACSCanto™; Becton Dickinson). Data were analyzed with FlowJo^®^ Software. For all samples the background MFI (i.e., autofluorescence of untreated and unlabeled cells) was subtracted. The percentage inhibition for each compound concentration was calculated relative to the positive control (i.e., untreated cells exposed to CXCL12^AF647^ only). Four parameter non-linear curve fitting (GraphPad Prism 9.0.2) was used to determine the IC_50_ value for binding inhibition.

### Calcium mobilization assay

The calcium (Ca^2+^) mobilization assay was described in detail before [24]. Briefly, U87.CD4.CXCR4 or U87.CD4.CCR5 cells (2 × 10^4^ cells per well in DMEM/10% FBS/0.01 M HEPES) were seeded in gelatin-coated (Sigma-Aldrich; 0.1% gelatin in DPBS) black-walled 96-well plates and incubated overnight at 37 °C and 5% CO_2_. The next day, cells were loaded with the fluorescent Ca^2+^ indicator Fluo-2 acetoxymethyl (AM) ester (4 μM final concentration; Abcam) and incubated at RT in the dark for 45 min. Then, cells were washed with assay buffer (see above) and subsequently incubated with different concentrations of compound for ~ 10 min prior to addition of 6.25 nM CXCL12 (in case of CXCR4) or 6.4 nM CCL5 (in case of CCR5). Fluctuations in intracellular Ca^2+^ levels were measured in real time by the FLIPR Tetra^®^ device (Molecular Devices) in all 96 wells simultaneously. The response (Relative Light Units, RLUs) over baseline was calculated (ScreenWorks 4.0^®^ software, Molecular Devices) by dividing the RLUs by a base line value measured just before addition of CXCL12 or CCL5, respectively. Inhibition of the Ca^2+^ response was determined taking into account negative (i.e., untreated cells without stimulation) and positive (i.e., untreated cells stimulated with either CXCL12 or CCL5) control samples.

### Pharmacokinetics

Pharmacokinetic analysis of plasma samples was performed at GVK BIO Sciences Pvt. Ltd. (Hyderabad, India). The pharmacokinetic properties of compound **18a** were determined by subcutaneous administration in male C57BL/6 mice. A 30 mg/kg dose was formulated as a solution in 23% hydroxypropyl-beta-cyclodextrin (HPBCD; w/v) in water. The dose volume was 3 mL/kg. Serial blood samples were collected at 0.5, 1, 2, 4, 6, 8 and 24 h post dosing (*n* = 3). Plasma concentration of compound **18a** was determined by a LC–MS/MS method. Chromatography was performed on a reverse phase column (Kinetex EVO, C18, 50 × 4.6 mm, 5 µ) at a flow rate of 1 mL/minute, using a gradient-elution method. Mobile Phase A was a 10 mM ammonium acetate solution with 0.1% formic acid in water, whereas mobile phase B was a mixture of acetonitrile and methanol (50:50). The gradient started at 5% B ramped up to 95% B over 1 min, held at 95% B for 1.2 min, and then ramped down to 5% B over 0.1 min, and held at 5% B for 1.2 min. The mass spectrometer was operated in positive-ionization mode and Telmisartan was used as internal standard. Analyst software was used for the data acquisition and chromatographic-peak integration. The C_max_, T_max_, AUC and half-life value (t_1/2_) were determined using standard non-compartmental pharmacokinetic methods. The concentration of compound **18a** in liver and lung tissue was determined by LC–MS/MS at Eurofins (Vergeze, France). Tissues harvested at the endpoint of the efficacy study (day 28 for liver, day 21 for lung), 2 h post the last compound **18a** dose, were processed. Around 250 mg of the lateral right liver lobe (*n* = 3) and ~ 120 mg of the caudal right lung lobe (*n* = 4) were homogenized in water and precipitated in acetonitrile containing Irbesartan as internal standard. Further analysis was performed via a similar method as described above. Data are expressed as mean ± sem.

### Ethical statement

The welfare of the animals was maintained in accordance with the general principles governing the use of animals in experiments of the European Communities (Directive 2010/63/EU) and Dutch legislation (The revised Experiments on Animals Act, 2014). This includes licensing of the project by the Central Committee on Animal Experimentation and approval and monitoring of the study by the The Netherlands Organisation for Applied Scientific Research (TNO) Animal Welfare Body (AWB).

### Liver fibrosis model

A preclinical mouse model for CCl_4_-induced liver fibrosis was performed at the Department of Metabolic Health Research, The Netherlands Organisation for Applied Scientific Research (TNO) (Leiden, The Netherlands). Fifty-two male C57BL/6J mice were matched on body weight prior to the study and divided into five groups (experimental study design, Supplementary Figure S1a). Starting at day 0, 10–12-week-old mice were intraperitoneally (i.p.) injected (three times a week) with mineral oil (group 1) or CCl_4_ (15% v/v in mineral oil, group 2–5) for a total of 28 days. At day 0, mice received subcutaneous treatment with compound **18a** (15 mg/kg/dose, twice daily (BID), group 3), or corresponding vehicle control (23% HPBCD, twice daily (BID), groups 1 and 2)], or received daily gavage with either an ALK5/ALK4 kinase inhibitor [SM 16 (45 mg/kg/dose, PO, QD, group 5) or its corresponding vehicle control (β-cyclodextrin sulfobutyl ether sodium salt or captisol, PO, QD, group 4)]. Plasma samples were collected by a tail vein bleed at day 7. At day 28, mice were sacrificed using CO_2_ and additional plasma samples and liver lobes were collected. As a primary endpoint collagen surface area was determined on cross-sections of the lateral left lobe by picro sirius red (PSR) staining. As a secondary endpoint collagen content (hydroxyproline) of the left median lobe was analyzed using a chromogenic assay according to the manufacturer’s instructions (QuickZyme Biosciences, Leiden, The Netherlands).

### Lung fibrosis model

A preclinical mouse model for bleomycin-induced lung fibrosis was performed at the Department of Metabolic Health Research, The Netherlands Organisation for Applied Scientific Research (TNO) (Leiden, The Netherlands). Thirty-eight male C57BL/6J mice were matched on body weight prior to the study and divided into three groups (experimental study design, Supplementary Figure S1b). At day 0, 10–12-week-old mice received a single oropharyngeal administration of bleomycin (0.04U/mouse) (group 2 and 3). Control mice (group 1) received phosphate buffered saline (PBS) instead. At day 0, bleomycin-treated mice received either compound **18a** (15 mg/kg/dose, subcutaneous, twice daily, group 3), or its corresponding vehicle control (23% HPBCD, subcutaneous, twice daily, group 2)]. PBS-treated mice received 23% HPBCD throughout the study (subcutaneous, twice daily, group 1). At day 21, mice were sacrificed under isoflurane anesthesia and plasma samples and lung lobes were collected. As a primary endpoint the histology lung fibrosis score (modified Ashcroft score [25]) was used based on a Masson’s Trichrome staining on cross-sections of the left lung lobe. As a secondary endpoint collagen content (hydroxyproline) was analyzed on the medial and accessory lobes by using a chromogenic assay according to the manufacturer’s instructions (QuickZyme Biosciences, Leiden, the Netherlands).

### Mouse CXCL12 plasma determination

CXCL12 plasma levels were determined using Bio-Plex Pro Mouse Chemokine SDF-1a/CXCL12 (Bio-Rad) according to the manufacturer’s protocol. Prior to the assay mouse plasma samples were centrifuged for 15 min at 1000×*g* at 4 °C to remove any particulates. A four-fold dilution series of mouse CXCL12, blank samples and 1:5 diluted plasma samples were prepared. A 1:1 mixture of magnetic beads (50 µL/well) and sample (50 µL/well) was added to the 96-well assay plate, which was then sealed and incubated (30 min at RT) on a plate shaker at 850 rpm protected from light. After washing three times with wash buffer 25 µL/well detection antibody was added and the plate was again incubated as described above. The plate was again washed three times (100 µL/well wash buffer) followed by the addition of streptavidin-PE (50 µL/well). The plate was sealed and incubated (10 min at RT) at 850 rpm protected from light. Finally, the plate was again washed three times and the magnetic beads were resuspended in fresh assay buffer (152 µL/well). The plate was sealed and shaken for an additional 30 s before being analyzed with the Bioplex 200 System. Data were analyzed with the Bio-Plex Manager Software.

### Quantitative reverse-transcription PCR (qRT-PCR) and nCounter analysis

Total RNA was prepared from snap-frozen liver tissue (the lateral and medial right lobes and the caudate lobe; Supplementary Figure S1a) or lung tissue (cranial right lobe; Supplementary Figure S1b) after homogenization on ice in TRIZol. For quantitative qRT-PCR, cDNA was made from 0.5 μg of RNA using the GoScript reverse transcription system (Promega) according to the manufacturer’s protocol. QRT-PCR was performed (for 40 cycles) with the Quantstudio3 Applied Biosystems (ABI) system using Sybr green (ABI) followed by melting curve analysis. Expression was calculated using the delta threshold cycle (ΔC_T_) method with *Gapdh* as reference gene (previously validated as stable house-keeping gene in the CCl_4_ model [26] and in the bleomycin model, current study, not shown). Forward (F) and reverse (R) primer sequences were (5’-3’): *Gapdh*: F: *ccgcatcttcttgtgcagt*, R: *gaatttgccgtgagtggagt*; *Col1a1*: F: *agcacgtctggtttggagag*, R: *gacattaggcgcaggaaggt*; *Ackr3*: F: *ctcaccgtcaggaaggcaaa*, R: *gccaggctctgcatagtcaa*; *Cxcr4*: F: *acggctgtagagcgagtgtt*, R: *ccgtcatgctccttagcttc*; *Golm1*: F: *gtgtgacgagcggatagagg*, R: *aattgggggctggaatctgg*; *Lgals3*: F: *cccaacgcaaacaggattgt*, R: *gaagcgggggttaaagtgga*; *Unc93b1*: F: *atggccattgtgcctctgtg*, R: *cgcgaagctcaagtggaaga*; *Slc15a3*: F: *ctctgaaagtgcccacctgt*, R: *aggtggactgcatctggaaat*; *Sec24d*: F: *cgtgttaccggaagcactgt*, R: *tcatgtacacaggcagcacc*; *Cxcl10*: F: *ctcatcctgctgggtctgag*, R: *tctttttcatcgtggcaatgatct*; and *Clec4n*: F: *tgaagggactatggtgtcagaaaa*, R: *agttctgctcactggtgctc*. Data are expressed as mean ± sem. For NanoString nCounter (NanoString, USA) analysis on liver RNA, a custom-designed panel of 52 fibrosis-related probes was used (the Nanostring probe list is shown in Supplementary Table 1). 100–500 ng of total RNA was hybridized according to the manufacturer’s instructions. Data were normalized by scaling to a panel of four housekeeping genes (i.e., *Hprt1*, *Actb*, *Ldh*, *Tbp*; probes are listed in Supplementary Table 1) with a coefficient of variation of < 5% between conditions used for comparison. The scaled counts were base log2-transformed and data were expressed as fold-change versus the reference condition (CCl_4_ + 23% HPBCD). Testing whether a contrast was significantly different was done by using a moderated *t*-test, as implemented in LIMMA. The resulting *P*-values were corrected for multiple testing with Benjamini–Hochberg to control the false discovery rate. *P* < 0.05 was considered as significant.

## Results

### Synthesis and in vitro pharmacology

**Fig. 1:**
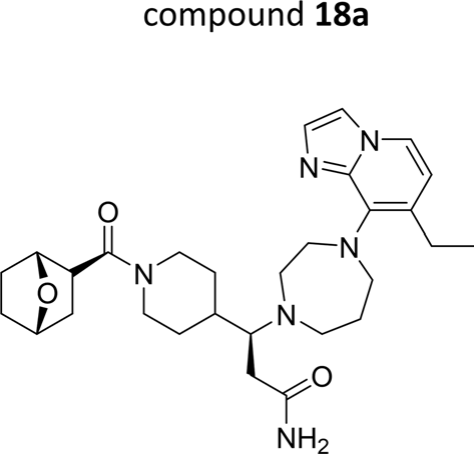
Chemical structure of compound 18a

The synthesis of the reference ACKR3 agonist was performed according to a literature procedure [21]. Since the synthesis involved the separation of a key intermediate by chiral chromatography, two final compounds were obtained which will be further referred to as compounds **18a** (Fig. 1) and **18b**. Although previously a difference in biological activity between both isomers was mentioned [21], no experimental data are available to support this. Therefore, a side-by-side comparison of compounds **18a** and **18b** was performed in various cell-based assays (Table 1). Binding of both isomers to hACKR3 was demonstrated in a CXCL12^AF647^ competition binding assay, whereby compound **18a** displayed a five-fold higher binding affinity (IC_50_ = 20.50 ± 6.34 nM) compared to compound **18b** (IC_50_ = 96.73 ± 10.08 nM; Table 1). Both isomers merely lacked binding affinity towards hCXCR4 (IC_50_ > 4 µM; Table 1). To confirm their agonistic activity, hACKR3-expressing cells were dose-dependently stimulated with compounds **18a** and **18b**, after which β-arrestin2 recruitment was measured. In this assay compound **18a** was also more potent than compound **18b** (EC_50_ = 3.16 ± 0.12 nM versus EC_50_ = 28.24 ± 5.44 nM; Table 1). Their potential antagonistic activity was further evaluated in an assay measuring the intracellular release of Ca^2+^ downstream the human chemokine receptors CXCR4 and CCR5, respectively. No antagonistic activity was observed (IC_50_ > 40 µM; Table 1), demonstrating specificity of the small molecules for hACKR3 over hCXCR4 and hCCR5. Taken together, these data indicate that compound **18a** is the most active isomer, and is identical to compound **18**, as published by scientists from Pfizer [21]. In contrast, compound **18b** behaves as the less active isomer. Therefore, subsequent pharmacokinetic and in vivo efficacy studies were performed with compound **18a**.

**Table 1:** In vitro pharmacology of compound18a and compound18b

Assay	Receptor	Read out	Compound18a	Compound18b
β-arrestin2 recruitment	hACKR3	EC_50_ (nM)	3.16 ± 0.12	28.24 ± 5.44
CXCL12^AF647^ competition binding	hACKR3	IC_50_ (nM)	20.50 ± 6.34	96.73 ± 10.08
CXCL12^AF647^ competition binding	hCXCR4	IC_50_ (nM)	> 4000	> 4000
Ca^2+^ mobilization	hCXCR4	IC_50_ (nM)	> 40,000	> 40,000
Ca^2+^ mobilization	hCCR5	IC_50_ (nM)	> 40,000	> 40,000

EC_50_ (β-arrestin2 recruitment) is the mean ± SD of two independent experiments. IC_50_ (hACKR3 binding) is the mean ± SD of three independent experiments

### Mouse plasma pharmacokinetics

Pharmacokinetic studies in mice previously demonstrated that compound **18a** was not orally bioavailable and subcutaneous administration was used to evaluate its efficacy in an isoproterenol-induced model of cardiac injury [21]. Prior to investigate the effect of compound **18a** in mouse models of liver and lung fibrosis, in vivo mouse pharmacokinetics at a dose of 30 mg/kg was investigated (Supplementary Table 2). After 0.25 h (T_max_), compound **18a** reached its maximal plasma concentration (C_max_) of 6116 ng/mL. Its elimination half-life (t_1/2_) was 5.2 h. The corresponding mean area under the plasma-concentration-versus-time profile (AUC) was 6699 ng/mL·h. Compound **18a** displayed an EC_50_ value of 3.16 nM (or 1.7 ng/mL) in the hACKR3 β-arrestin2 recruitment assay. Taking into account that the free fraction of compound **18a** in mouse plasma was determined previously at 34% [21], this corresponds to an EC_50_ value of 5.2 ng/mL. The plasma levels that are reached after subcutaneous administration of compound **18a** at a dose of 30 mg/kg largely exceeded this target value for up to 8 h after administration, but dropped below this target value at 24 h, and hence, this dose was administered twice daily (2 × 15 mg/kg) for subsequent in vivo experiments.

### In vivo liver fibrosis study

**Fig. 2:**
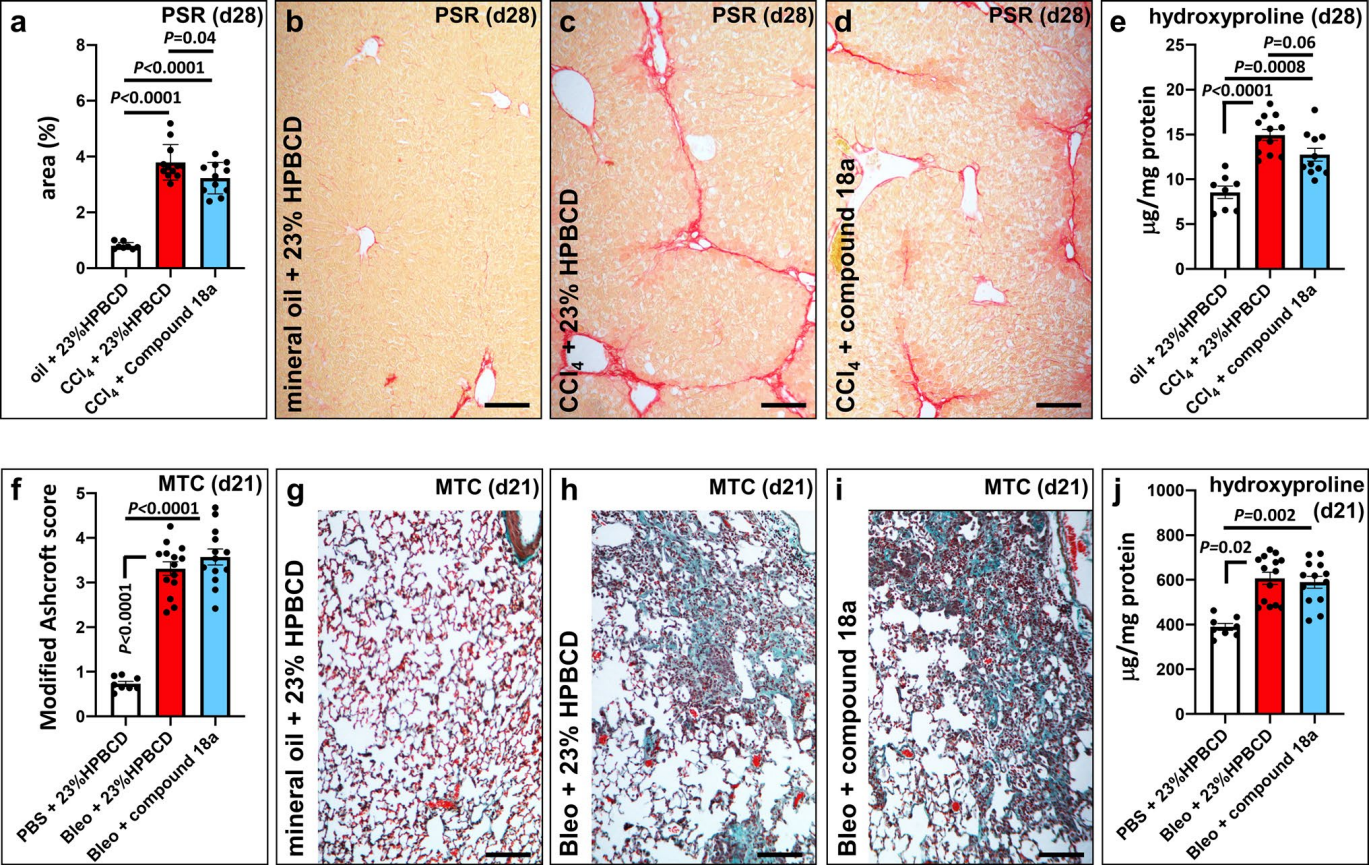
Compound 18a attenuates liver, but not lung fibrosis. **a–d** Diagram (*a*) showing quantification of picro sirius red (PSR) area at day (d)28, expressed as a percentage versus tissue area ± sem, and representative pictures of PSR-stained liver cross-sections of mice treated with mineral oil + 23% HPBCD (*b*; white in *a*; group 1; *n* = 8), CCl_4_ + 23% HPBCD (*c*; red in *a*; group 2; *n* = 12) or CCl_4_ + compound **18a** (*d*; blue in *a*; group 3; *n* = 11). **e** Diagram showing quantification of hydroxyproline content at d28, expressed per weight of protein ± sem. **f–i** Diagram (*f*) showing quantification of Masson’s Trichrome (MTC) area at d21, expressed as modified Ashcroft score ± sem, and representative pictures of MTC-stained lung cross-sections of mice treated with mineral oil + 23% HPBCD (*g* white in *f*; group1; *n* = 8), bleomycin + 23% HPBCD (*h*; red in *f*; group 2; *n* = 14) or bleomycin + compound **18a** (*i*; blue in *f*; group 3; *n* = 13). **j** Diagram showing quantification of hydroxyproline content at d21, expressed per weight of protein ± sem. Quantitative data in *a*, *e* and *f* were plotted and analyzed (using Graphpad Prism; version 9.0.1) by one-way ANOVA with Tukey’s post-hoc test and in *j* by Kruskal–Wallis with Dunn’s post-hoc test. Scale bars in *b-d* and *g-i* represent 100 μm. Bleo: bleomycin; CCl_4_: carbon tetrachloride; HPBCD: hydroxypropyl-beta-cyclodextrin. Figure composition was made in Microsoft Powerpoint

An overview of the study design is given in Supplementary Figure S1a. Liver fibrosis was induced in male C57BL/6J mice by i.p. injection of CCl_4_ for up to four weeks. Starting at day 0, compound **18a** (15 mg/kg/dose) or its corresponding vehicle control (23% HPBCD) was administered twice daily (BID). As a positive reference treatment condition, other mice received daily gavage with either an ALK5/ALK4 kinase inhibitor [22] [SM 16 (45 mg/kg/dose)] or its corresponding vehicle control (captisol). One animal in the CCl_4_-induced vehicle (23% HPBCD) control group was found dead in the cage at the end of the study (day 28), two animals in the compound **18a** treatment group died during CCl_4_ injection at day 21 and 26, respectively, and one animal in the SM 16 treatment group was found dead in the cage at day 19. Over the course of the study, CCl_4_ treatment did not significantly change the body weight of the mice, but led to an increased liver weight per body weight that was not affected by compound **18a** treatment (Supplementary Figure S2a, b). In agreement with previous observations in liver ECs[17], expression of *Cxcr4* on total liver tissue was increased, while *Ackr3* expression remained unchanged after chronic exposure to CCl_4_ (Supplementary Figure S3a, b). Development of liver fibrosis was confirmed in CCl_4_-treated mice by significant upregulation of *Col1a1* expression (Supplementary Figure S3c) and increased deposition of fibrillar collagen matrix mostly along the axes connecting the venules (bridging fibrosis) in liver samples determined by both PSR staining and quantification of hydroxyproline content (Fig. 2a–c, e). At the end of the study, compound **18a** treatment of CCl_4_-induced mice resulted into modestly, but significantly, reduced collagen levels (mostly evident from incomplete bridging) when assessed by PSR staining (Fig. 2a, c, d; *P* = 0.04, by one-way ANOVA and Tukey’s post-hoc test) and a near significant reduction of hydroxyproline levels (Fig. 2e; *P* = 0.06, by one-way ANOVA and Tukey’s post-hoc test). Mice receiving the positive reference control treatment with SM 16 showed significantly attenuated liver fibrosis compared to its vehicle control, as shown by reduced PSR-positive area and hydroxyproline content (Supplementary Figure S4a,b; *P* < 0.0001 and *P* = 0.004, respectively, by one-way ANOVA and Tukey’s post-hoc test).

### In vivo lung fibrosis study

An overview of the study design is given in Supplementary Figure S1b. Ten to twelve-week-old C57BL/6J mice received a single oropharyngeal administration of bleomycin (0.04U/mouse) to induce lung fibrosis. Two animals in the compound **18a** treatment group and one animal in the bleomycin-induced control group were prematurely sacrificed at day 13, 17 and 21, respectively due to severe loss of body weight (> 25% of the starting weight). At the end of the study (day 21), lung weight per body weight was significantly increased in bleomycin-treated mice (independent of the treatment condition), whereas body weight itself was only slightly reduced (Supplementary Figure S2c, d). Compound **18a** (15 mg/kg/dose) or its corresponding vehicle control (23% HPBCD) was subcutaneously administered starting at day 0. As expected, bleomycin-induction led to a significant increase in collagen levels confirmed by significantly increased Masson’s Trichrome staining and hydroxyproline levels (Fig. 2f–h, j). However, treatment of bleomycin-induced mice with compound **18a** did not counteract the development of fibrosis (Fig. 2f, h–j).

### Mouse CXCL12 plasma determination

**Fig. 3:**
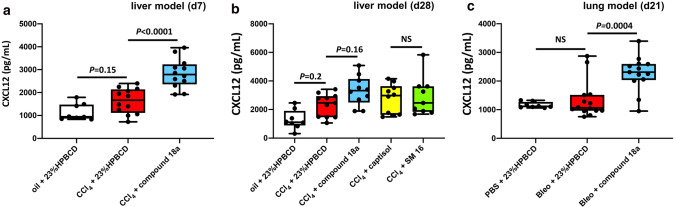
Compound 18a increases mouse CXCL12 plasma levels. **a** At day 7 of the liver injury model compound **18a** significantly increased mouse CXCL12 levels in the plasma of CCl_4_-treated mice. **b** At the end of the liver injury model day (d)28, compound **18a** treatment led to increased CXCL12 levels in CCl_4_-treated animals, whereas SM 16 (ALK5/ALK4 kinase inhibitor) did not. **c** At the end of the preclinical lung injury model, in vivo administration of compound **18a** increased mouse CXCL12 plasma levels compared to vehicle (23% HPBCD) control mice. Quantitative data in *a*-*c* were plotted as box plots showing the maximum and minimum values and analyzed (using Graphpad Prism; version 9.0.1) by one-way ANOVA with Tukey’s post-hoc test. NS; no significant difference, Bleo: bleomycin; CCl_4_: carbon tetrachloride; HPBCD: hydroxypropyl-beta-cyclodextrin. Figure composition was made in Microsoft Powerpoint

Mouse plasma samples were collected at the end of both the liver fibrosis (day 28) and lung fibrosis (day 21) study. Plasma was also collected at day 7 of the liver fibrosis model. CXCL12-levels were determined for all animals, with exception of the animals that died or were prematurely sacrificed during the study. Baseline CXCL12 plasma levels (~ 1 ng/mL; Fig. 3) were in line with previously determined values [21]. At day 7 and 28 of the liver fibrosis model, CCl_4_ treatment tended to increase plasma CXCL12 (Fig. 3a, b). Treatment of the mice with compound **18a** further increased CXCL12-levels up to ~ threefold at day 7 and tended to further increase CXCL12-levels compared to baseline (Fig. 3). Of note, in case mice were treated with SM 16, no further increase of plasma CXCL12 was observed, indicating the specific contribution of compound **18a** to increased CXCL12-levels (Fig. 3b). In the lung fibrosis model, a single administration of bleomycin did not induce elevated CXCL12-levels by itself at day 21, however, compound **18a** treatment did (Fig. 3c). Taken together, in both in vivo models compound **18a** did increase plasma CXCL12-levels, strongly suggesting in vivo interaction with its target receptor hACKR3. Hence, we confirmed the in vivo pharmacodynamic effect of compound **18a** [21].

### Organ-dependent differential effects of compound 18a on fibrosis

**Fig. 4:**
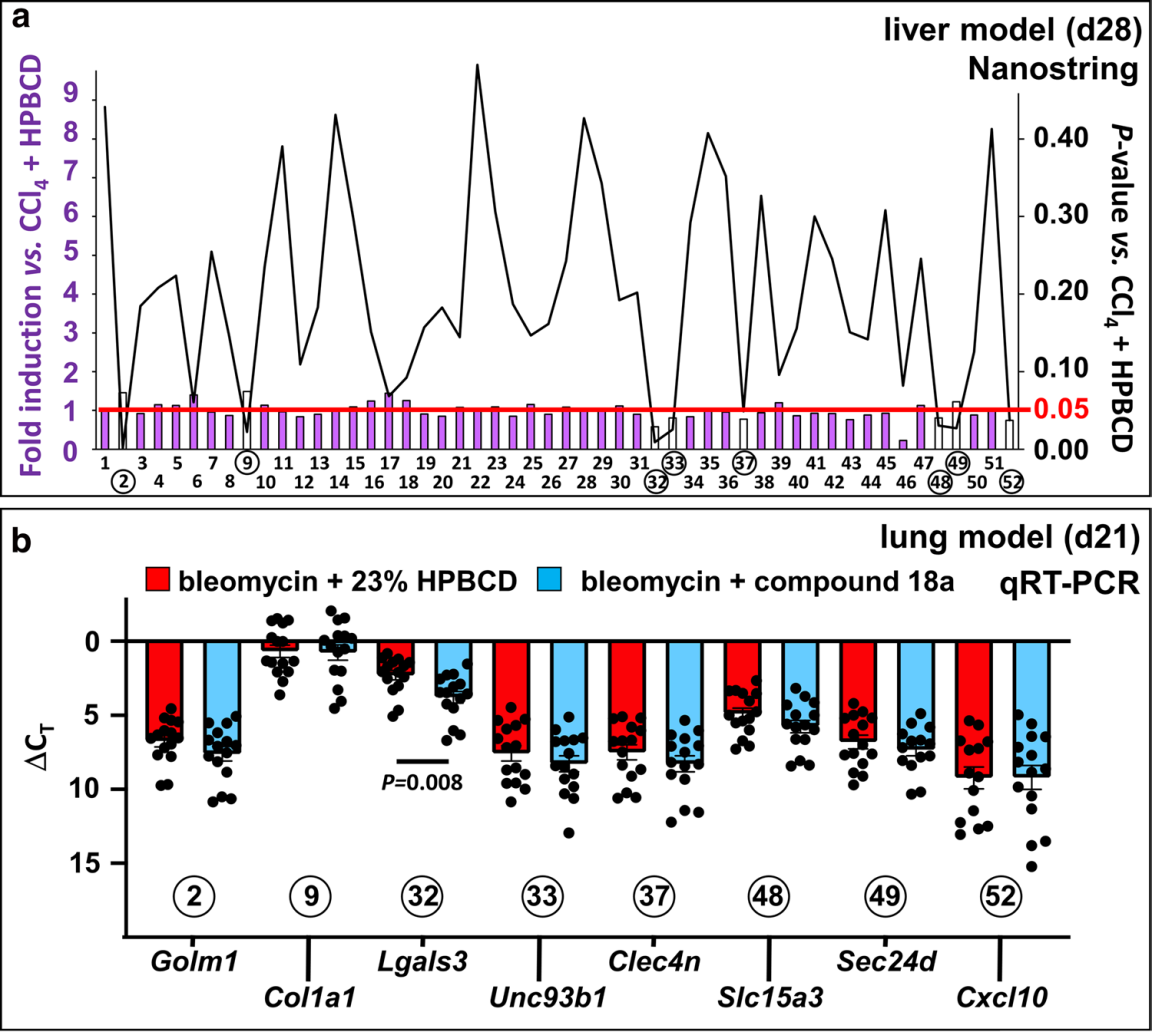
Compound 18a differentially affects fibrosis-associated gene expression in liver and lungs. **a** Diagram showing the effect of compound **18a** on the mRNA expression of a panel of 52 fibrosis-associated genes (corresponding genes and numerical data are shown in Supplementary Table 1), measured by nCounter analysis and expressed as fold-difference of compound-treated versus vehicle-treated mice after 28 days (d) of exposure to CCl_4_. Significantly (*P* < 0.05 indicated by the red horizontal line) altered genes are shown as transparent bars. **b** Diagram showing mRNA expression of 8 fibrosis-associated genes [corresponding to those significantly altered in (*a*)] at day (d)21 after induction of lung fibrosis measured by qRT-PCR and expressed as delta threshold cycle (ΔC_T_) ± sem of mice treated with bleomycin + 23% HPBCD (red; group 2; *n* = 15) or bleomycin + compound **18a** (blue; group 3; *n* = 15). All quantitative data were plotted and analyzed (using Graphpad Prism; version 9.0.1) by unpaired Student’s *t*-test. CCl_4_: carbon tetrachloride; HPBCD: hydroxypropyl-beta-cyclodextrin. Figure composition was made in Microsoft Powerpoint

Given the modest but significant attenuating effect on fibrosis in the liver and the lack of effect in the lung, we first examined whether this organ-dependent response to compound **18a** was due to a difference in tissue distribution. Determination of the concentration of compound **18a** in liver and lung tissue homogenates 2 h after the last subcutaneous compound injection revealed no significant difference (ng/g tissue: 2528 ± 511 in liver (day 28) versus 1703 ± 54 in lung (day 21), *P* = 0.19 by unpaired Student’s *t*-test). Next, we investigated whether compound **18a** might have a differential effect on a custom-designed panel of 52 fibrosis-related genes in fibrotic livers versus lungs. While compound **18a** had a significant effect on the expression of 8 out of 52 genes in CCl_4_-challenged livers (Fig. 4a and Supplementary Table 1), only one of these genes was similarly affected in bleomycin-exposed lungs (Fig. 4b) compared to the corresponding vehicle-treated reference conditions. Altogether, the organ-specific effect of compound **18a** was likely due to a differential effect on fibrotic gene expression rather than a difference in liver versus lung concentration.

## Discussion

Several recent studies reported the contribution of ACKR3 activity (agonism) to tissue regeneration and amelioration of fibrosis in diverse tissues including liver and lung [17, 18, 27]. In this study, we further embarked upon this protective role of ACKR3 activity by evaluating an ACKR3-specific small molecule agonist (compound **18a**) in preclinical mouse models of lung and liver fibrosis. Compound **18a** was previously shown to protect mice from cardiac fibrosis in an isoproterenol-induced model of cardiac injury [21] and was endowed with a pharmacokinetic profile that allowed in vivo administration. Using a β-arrestin2 recruitment assay compound **18a** was shown to be a highly potent ACKR3 agonist. Of interest, compound **18a** did not inhibit the activation of CXCR4, another chemokine GPCR that shares the chemokine ligand CXCL12 with ACKR3. Therefore, applying compound **18a** in the in vivo lung and liver fibrosis models allowed us to evaluate the anti-fibrotic effect of pure ACKR3 agonism in the absence of CXCR4 antagonism, which has never been addressed before. Indeed, in previous studies a functional role for ACKR3 activity in tissue regeneration and prevention of fibrosis was established based on conditional knockouts of *Ackr3* in the endothelial compartment of injured liver and lung, as well as based on the in vivo administration of TC14012, a peptide-based ACKR3 agonist with only modest potency [17, 18, 27]. Since TC14012 is also a well-established and highly potent CXCR4 antagonist, it cannot be excluded that the anti-fibrotic effect previously observed for TC14012 results from a beneficial combined effect of ACKR3 agonism and CXCR4 antagonism. In accordance, in this previous study with TC14012, it was shown that the expression of *Ackr3* in liver ECs was temporarily increased early during the chronic CCl_4_ model and then returned to baseline levels in the longer term. The expression kinetics of *Cxcr4* was however different, since its expression only started to rise after the peak of *Ackr3* expression [17]. This suggests that the impact of ACKR3 agonism is rather important in the early stages, while CXCR4 antagonism becomes more important in the chronic phase of liver fibrosis. This may explain the rather modest effect on liver fibrosis of the selective ACKR3 agonist we tested here, compared to TC14012 with its combined (opposite) activity on both receptors. Given the low potency of TC14012 as an ACKR3 agonist, it might be worthwhile to test a combination of TC14012 with the more potent compound **18a** in order to boost the agonistic effect on ACKR3. Furthermore, the fact that the ACKR3 agonist compound **18a** increased the bioavailability of CXCL12 (which can act as an agonist on the CXCR4 receptor) may even have counterbalanced the beneficial effect on liver fibrosis.

In both in vivo mouse models, fibrosis was successfully induced by either chronic CCl_4_ treatment (i.e., liver fibrosis) or single application of bleomycin (i.e., lung fibrosis) compared to control mice. As mentioned above, treatment with compound **18a** led to a significant increase of plasma CXCL12 levels, in line with the CXCL12 scavenging function of ACKR3. This effect on plasma CXCL12 strongly indicates the genuine pharmacodynamic effect of compound **18a** in both models. Nevertheless, we did, however, only observe an effect of compound **18a** treatment on the reduction of liver and not lung fibrosis. This tissue-specific effect may have different reasons. One potential explanation is that the expression pattern of *Ackr3* varies across the endothelium in various tissues with lung ECs having a significantly higher expression than liver or brain endothelium [28, 29]. The higher expression in lung ECs (together with local availability of CXCL11/CXCL12) may already cause a maximal effect, so that the additive effect of a receptor agonist is more limited than in tissues with lower *Ackr3* expression. Another indication that a compound can have differential effects in different tissues is the fact that while the anti-fibrosis process in the liver might rather benefit from an antagonistic effect on CXCR4, the opposite effect on CXCR4 may be required to counter fibrosis in the heart, as suggested earlier [21]. It is unknown how and to what extent CXCR4 signaling affects lung fibrosis and whether the lack of an inhibitory effect on CXCR4 is one of the reasons for the failure of compound **18a** to attenuate lung fibrosis. Thirdly, the differential effect may be due to the fact that in the in vivo models compound **18a** may have reached significantly higher levels in liver compared to lung, however our pharmacokinetic studies in tissues have ruled out this reasoning. Finally, the differential effect of compound **18a** may be related to a context-dependent effect on the molecular players involved in fibrogenesis. To explore this possibility, we quantified the effect of the compound on the expression of a custom-designed panel of 52 fibrosis-associated genes. We found that while 8 of the 52 (~ 15%) genes were significantly affected by compound **18a** in the liver, only 1 of these 8 genes (i.e., *Lgals3*) was similarly lowered in its expression in lung tissue, the latter likely being insufficient to counter fibrosis. While the broader effect of compound **18a** on fibrosis-associated genes in the liver likely contributes to its differential ability to affect fibrosis, it remains to be determined which of these gene alterations is causally involved in attenuation of fibrosis in the liver. Intriguingly, although PSR staining and hydroxyproline assays both indicated a lower collagen content in livers upon compound **18a** treatment, the expression levels of *Col1a1* were slightly increased by the compound. This raises the possibility that compound **18a** may lower collagen content by promoting degradation rather than inhibiting synthesis of collagen or that it may affect post-translational modifications. Interestingly, one of the genes significantly lowered by our ACKR3 agonist encoded CXCL10, a ligand for CXCR3. Blocking or knocking out *Cxcl10* in mice has been shown to attenuate CCl_4_-induced liver fibrosis [30]. CXCR3 and ACKR3 have a common ligand, i.e., CXCL11, hence an ACKR3 agonist may have a complex effect on the bioavailability of ligands for the CXCR3 receptor.

Recent single-cell RNA sequencing on healthy human liver tissue revealed that modest *ACKR3* expression is mostly seen in the endothelial and mesenchymal (vascular smooth muscle and fibroblast) compartment, to some extent in epithelial cell types (mostly hepatocytes) and not in leukocyte lineages [31] (Supplementary Figure S5a, b). Within the endothelial compartment, *ACKR3* is expressed in multiple subtypes, including *CLEC4G*^+^/*CLEC4M*^+^ LSECs and a cluster that expresses angiocrine markers (*RSPO3* and *WNT2*) that have been associated with pericentral liver ECs (Supplementary Figure S5c, d) [32–34]. Interestingly, expression of *ACKR3* was mostly upregulated in the latter cluster in livers with cirrhosis, an advanced stage of liver fibrosis (Supplementary Figure S5c, d) [31]. A population of self-renewing hepatocyte progenitor cells has been described within the pericentral area that responds to Wnt2 angiocrine signals from central vein ECs [33]. Therefore, it remains to be seen whether the pro-regenerative balance induced by ACKR3 agonism is also in part related to the central vein ECs, in addition to its reported similar role in LSECs [17]. Whether ACKR3 agonism on mesenchymal cells also contributes to its pro-regenerative/anti-fibrotic effect remains to be determined.

ACKR3 in ECs has also been shown to mediate proliferation and angiogenesis [35]. This proangiogenic activity in the heart was associated with cardio-protection during acute myocardial infarction [36]. While in the heart, a positive correlation has been observed between angiogenesis and cardio-protection, the effect of angiogenesis in the injured liver on fibrosis is not unequivocal [37, 38]. On the one hand, fibrosis has been associated with increased angiogenesis leading to the use of anti-angiogenic strategies to conquer liver fibrosis [37, 38]. On the other hand, angiogenesis is also needed to support liver repair after (fibrotic) damage suggesting that stimulation of angiogenesis would be beneficial for liver repair [38]. It remains to be determined whether ACKR3 agonism in the liver, like in the heart [36], has a pro-angiogenic effect and whether this has a beneficial or rather detrimental effect on liver fibrosis.

Another way in which ECs can contribute to fibrosis is by undergoing a transition to collagen-producing mesenchymal cells, a process called endothelial-to-mesenchymal transition (EndoMT) [39]. ACKR3 overexpression in pulmonary arterial ECs attenuated transforming growth factor (TGF) β1-induced EndoMT and the ACKR3 agonist TC14012 reduced mesenchymal marker expression and fibrosis in the lung of mice exposed to TGFβ1-encoding adenovirus [27]. Whether the effect of TC14012 on liver fibrosis was also related to EndoMT was not documented. While the absence of an effect on pulmonary fibrosis by compound **18a** seems to rule out the possibility of a significant effect on EndoMT in the lung, it remains to be determined whether the attenuating effect of compound **18a** on liver fibrosis is to some extent related to reducing EndoMT. The latter may be unlikely as it has been shown that the liver, unlike other organs, only shows limited signs of EndoMT during fibrosis development [39, 40].

In conclusion, whereas the TC14012 ACKR3 agonist successfully attenuated fibrosis in liver and lungs, the ACKR3 agonist compound **18a** was not effective in reducing lung fibrosis, but modestly attenuated fibrosis in the liver. It remains to be determined whether this differential effect of both agonists is due to a different mechanism of action, *e.g.* the simultaneous antagonism of CXCR4 by TC14012 versus the lack thereof in case of compound **18a**. Furthermore, while our studies suggest that attenuation of liver fibrosis may be related to affecting the expression of fibrosis-related genes in an organ-specific manner, the underlying causal mechanisms by which compound **18a** attenuated liver fibrosis remain to be determined in future studies. Additional experiments may also address the question whether combining both agonists may cause a synergistic effect on tackling fibrosis in the liver.

### Supplementary Information

Below is the link to the electronic supplementary material.

Supplementary file1 Supplementary Fig. S1 Experimental design (a) Table (left) showing the different treatment conditions and timing of the experiments related to the liver fibrosis model. Schematic drawing (right) of the different liver lobes and their use. (b) Table (left) showing the different treatment conditions and timing of the experiments related to the lung fibrosis model. Schematic drawing (right) of the different lung lobes and their use. PK: pharmacokinetics; SC: subcutaneous; PO: per oral; BID: twice daily; QD: once daily; HPBCD: hydroxypropyl-beta-cyclodextrin; captisol: β-Cyclodextrin sulfobutyl ether, sodium salt; CCl4: carbon tetrachloride; PSR: picro Sirius red; MTC: Masson’s Thrichrome. Figure composition was made in Microsoft Powerpoint. (TIF 4382 KB)

Supplementary file2 Supplementary Fig. S2 Weight analysis (a-d) Diagrams showing body weight (a) or liver weight relative to body weight (BW) (b) at day (d)28 after induction of liver fibrosis; and body weight (c) or lung weight relative to body weight (BW) (d) at d21 after induction of lung fibrosis ± sem of mice treated with mineral oil or PBS + 23% HPBCD (white; group 1; n=8 in a-d), CCl4/Bleo + 23% HPBCD (red; group 2; n=11 in a,b, n=14 in c,d) or CCl4/Bleo + compound 18a (blue; group 3; n=10 in a,b, n=13 in c,d). All quantitative data were plotted and analyzed (using Graphpad Prism; version 9.0.1) by one-way ANOVA with Tukey post-hoc test. Bleo; bleomycin; CCl4: carbon tetrachloride; HPBCD: hydroxypropyl-beta-cyclodextrin. Figure composition was made in Microsoft Powerpoint. (TIF 1768 KB)

Supplementary file3 Supplementary Fig. S3 mRNA analysis (a-c) Diagrams showing mRNA expression of Cxcr4 (a), Ackr3 (b) and Col1a1 (c) at day (d)28 after induction of liver fibrosis expressed as delta threshold cycle (±CT) ± sem of mice treated with mineral oil + 23% HPBCD (white; group 1; n=7-8) or CCl4 + 23% HPBCD (red; group 2; n=11). All quantitative data were plotted and analyzed (using Graphpad Prism; version 9.0.1) by unpaired Student’s t-test. CCl4: carbon tetrachloride; HPBCD: hydroxypropyl-beta-cyclodextrin. Figure composition was made in Microsoft Powerpoint. (TIF 1481 KB)

Supplementary file4 Supplementary Fig. S4 Analysis of reference treatment condition in the liver fibrosis model (a,b) Diagrams showing quantification of picro sirius red (PSR) area (a), expressed as a percentage versus tissue area ± sem, and quantification of hydroxyproline content (b) at day (d)28, expressed per weight of protein ± sem in mice treated with mineral oil + 23% HPBCD (white; group 1; n=8), CCl4 + captisol (yellow; group 4; n=10) or CCl4 + SM 16 (green; group 5; n=9). All quantitative data were plotted and analyzed (using Graphpad Prism; version 9.0.1) by one-way ANOVA with Tukey’s post-hoc test. CCl4: carbon tetrachloride; HPBCD: hydroxypropyl-beta-cyclodextrin; captisol: β-Cyclodextrin sulfobutyl ether, sodium salt. Figure composition was made in Microsoft Powerpoint. (TIF 1433 KB)

Supplementary file5 Supplementary Fig. S5 Expression of ACKR3 mRNA at single-cell level in healthy and cirrhotic human livers (a,b) Diagrams showing cell cluster analysis by t-Distributed Stochastic Neighbor Embedding (t-SNE; a), revealing 12 cell types represented in human healthy or cirrhotic liver single-cell suspensions and violin plots (b) reporting expression of ACKR3 in each cell cluster in healthy (gray) and cirrhotic livers (purple), revealing expression mostly in the endothelial (EC), mesenchymal (mes) and hepatocyte (hep) clusters. (c,d) Diagrams showing cell cluster analysis by t-SNE (c), revealing 7 EC subtypes represented in human healthy or cirrhotic liver single-cell suspensions and violin plots (d) reporting expression of ACKR3 in each cell cluster in healthy (gray) and cirrhotic livers (purple), revealing expression in most subtypes, including CLEC4G+/CLEC4M+ liver sinusoidal ECs (LSECs; orange) and WNT2+/RSPO3+ ECs from the pericentral area (green). Note the significant increase in the latter cluster in cirrhotic versus healthy livers. MP: mononuclear phagocyte; pDC; plasmacytoid dendritic cell; ILC: innate lymphoid cell; EC: endothelial cell. Data were retrieved from the publicly available gene browser link provided by Ramachandran et al.[31]. Figure composition was made in Microsoft Powerpoint. (TIF 8156 KB)

Supplementary file6 (DOCX 30 KB)

Supplementary file7 (DOCX 14 KB)

## Data Availability

All data generated or analyzed during this study are included in this manuscript and its supplementary information files.

## Abbreviations

ACKR3: Atypical chemokine receptor 3
CCR5: CC chemokine receptor 5
CXCR3: CXC chemokine receptor 3
CXCR4: CXC chemokine receptor 4
CXCR7: CXC chemokine receptor 7
CXCL11: CXC chemokine ligand 11
CXCL12: CXC chemokine ligand 12
CCl_4_: Carbon tetrachloride
GPCR: G protein-coupled receptor
HPBCD: Hydroxypropyl-beta-cyclodextrin
I-TAC: Interferon-inducible T-cell alpha chemoattractant
LSEC: Liver sinusoidal endothelial cell
PCEC: Pulmonary capillary endothelial cell
PE: Phycoerythrin
PSR: Picro sirius red
SDF-1α: Stromal cell derived factor 1α
