# Supplementary figures and images for: Stimulation of the atypical chemokine receptor 3 (ACKR3) by a small-molecule agonist attenuates fibrosis in a preclinical liver but not lung injury model
Source: Cell Mol Life Sci. 2022 May 13;79(6):293. doi: 10.1007/s00018-022-04317-y (PMC9106635; doi:10.1007/s00018-022-04317-y)

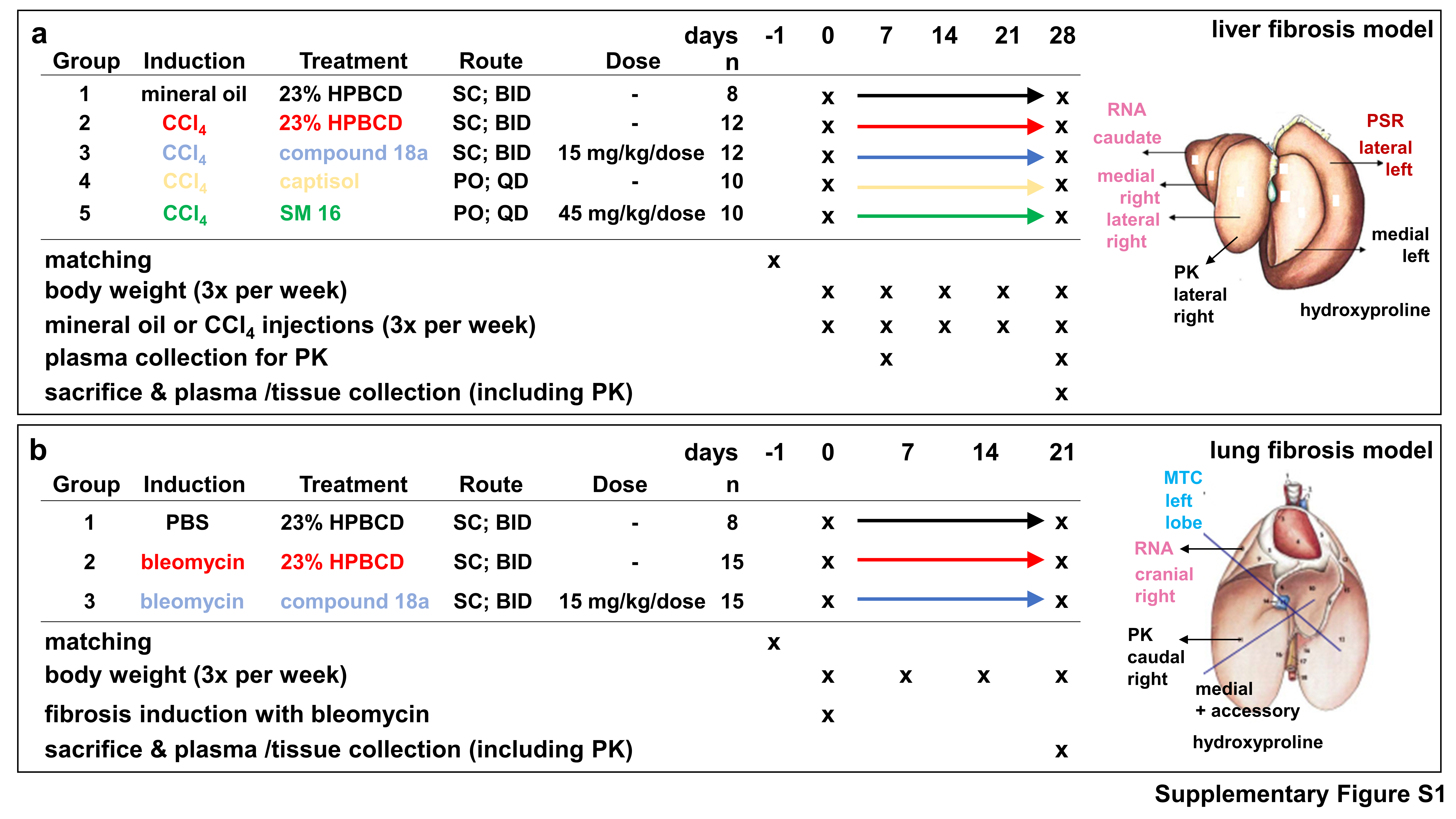

Supplement: Supplementary file 1 — Supplementary file1 Supplementary Fig. S1 Experimental design (a) Table (left) showing the different treatment conditions and timing of the experiments related to the liver fibrosis model. Schematic drawing (right) of the different liver lobes and their use. (b) Table (left) showing the different treatment conditions and timing of the experiments related to the lung fibrosis model. Schematic drawing (right) of the different lung lobes and their use. PK: pharmacokinetics; SC: subcutaneous; PO: per oral; BID: twice daily; QD: once daily; HPBCD: hydroxypropyl-beta-cyclodextrin; captisol: β-Cyclodextrin sulfobutyl ether, sodium salt; CCl4: carbon tetrachloride; PSR: picro Sirius red; MTC: Masson’s Thrichrome. Figure composition was made in Microsoft Powerpoint. (TIF 4382 KB) [file 18_2022_4317_MOESM1_ESM.tif]

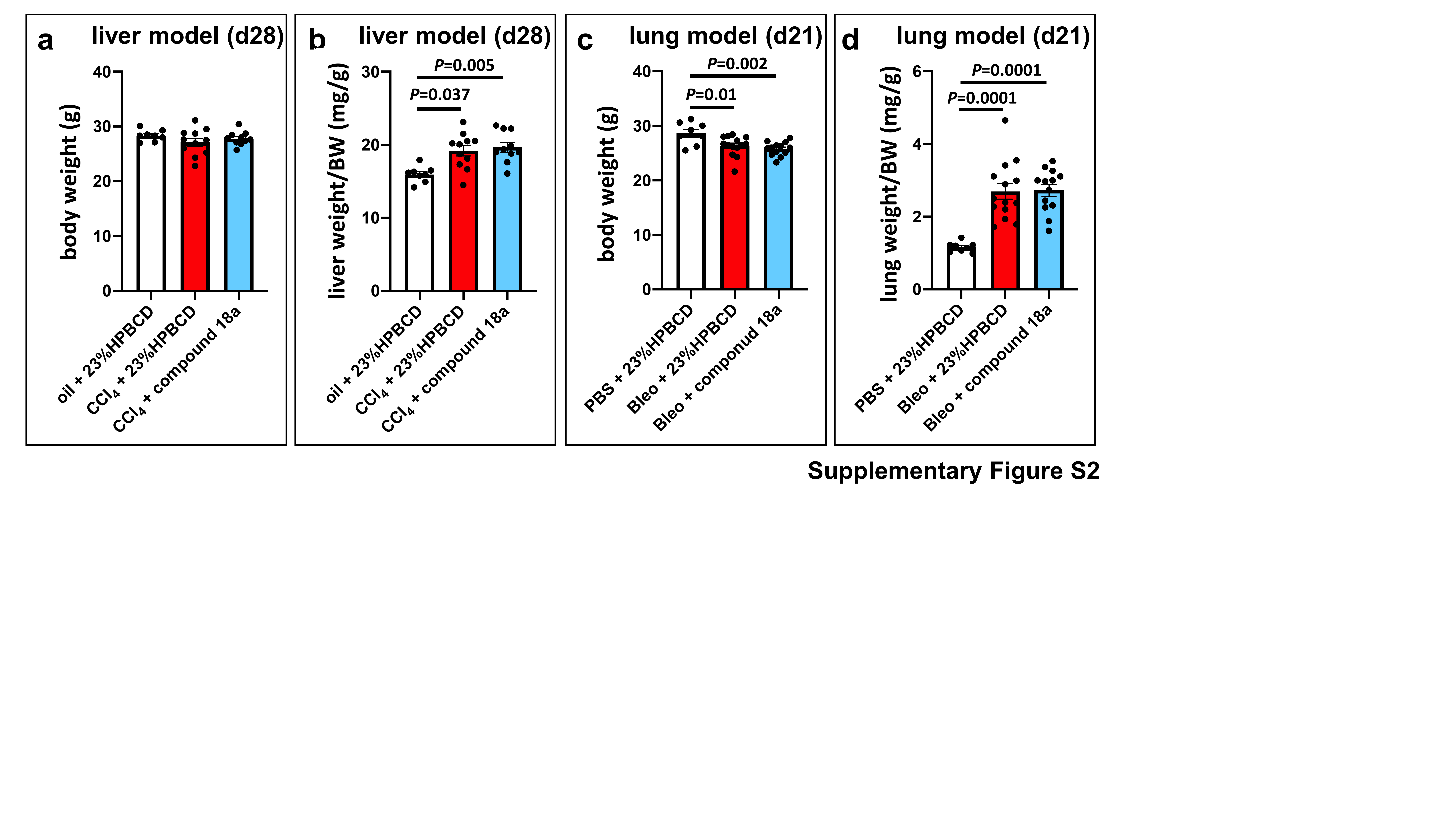

Supplement: Supplementary file 2 — Supplementary file2 Supplementary Fig. S2 Weight analysis (a-d) Diagrams showing body weight (a) or liver weight relative to body weight (BW) (b) at day (d)28 after induction of liver fibrosis; and body weight (c) or lung weight relative to body weight (BW) (d) at d21 after induction of lung fibrosis ± sem of mice treated with mineral oil or PBS + 23% HPBCD (white; group 1; n=8 in a-d), CCl4/Bleo + 23% HPBCD (red; group 2; n=11 in a,b, n=14 in c,d) or CCl4/Bleo + compound 18a (blue; group 3; n=10 in a,b, n=13 in c,d). All quantitative data were plotted and analyzed (using Graphpad Prism; version 9.0.1) by one-way ANOVA with Tukey post-hoc test. Bleo; bleomycin; CCl4: carbon tetrachloride; HPBCD: hydroxypropyl-beta-cyclodextrin. Figure composition was made in Microsoft Powerpoint. (TIF 1768 KB) [file 18_2022_4317_MOESM2_ESM.tif]

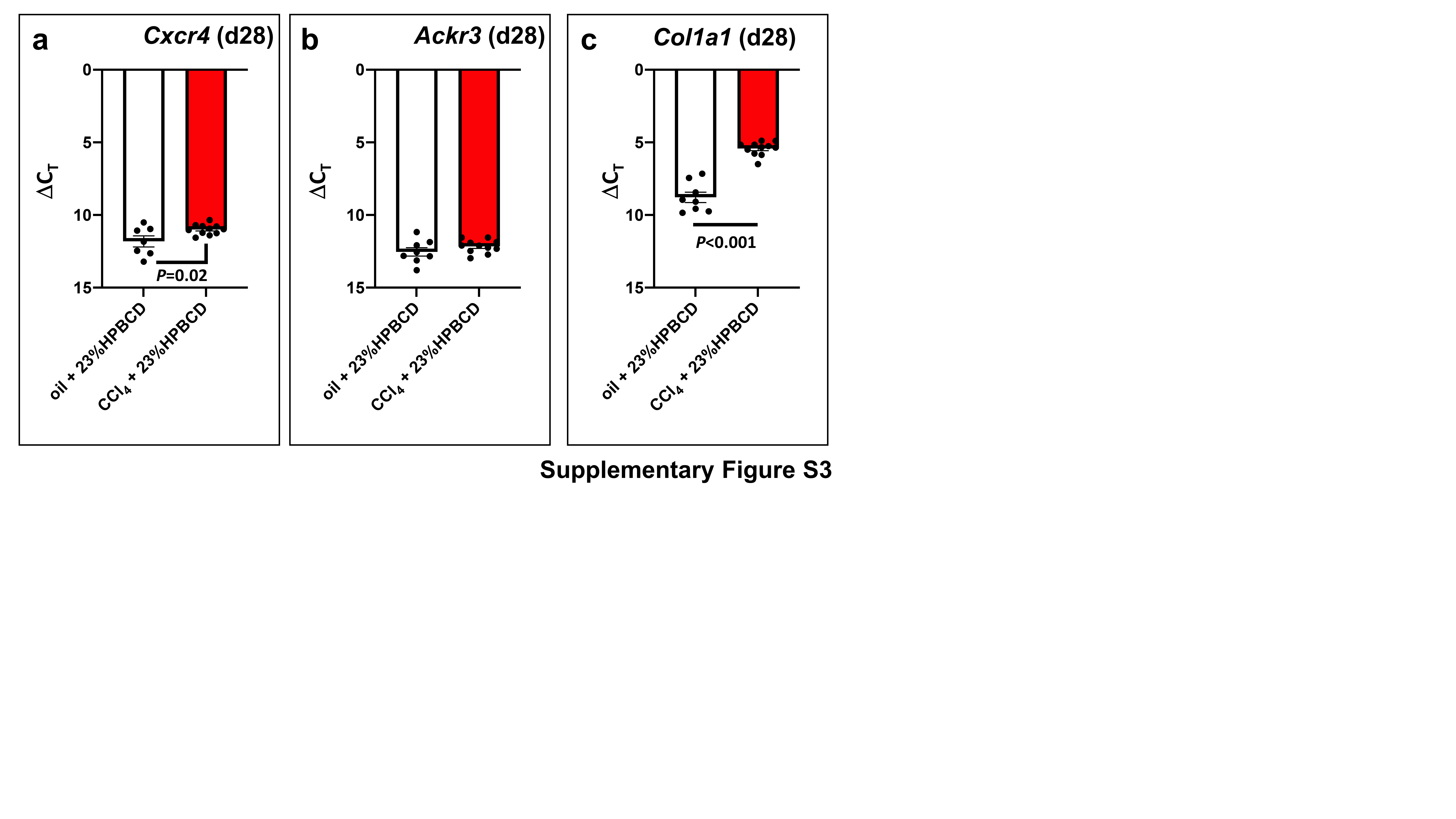

Supplement: Supplementary file 3 — Supplementary file3 Supplementary Fig. S3 mRNA analysis (a-c) Diagrams showing mRNA expression of Cxcr4 (a), Ackr3 (b) and Col1a1 (c) at day (d)28 after induction of liver fibrosis expressed as delta threshold cycle (±CT) ± sem of mice treated with mineral oil + 23% HPBCD (white; group 1; n=7-8) or CCl4 + 23% HPBCD (red; group 2; n=11). All quantitative data were plotted and analyzed (using Graphpad Prism; version 9.0.1) by unpaired Student’s t-test. CCl4: carbon tetrachloride; HPBCD: hydroxypropyl-beta-cyclodextrin. Figure composition was made in Microsoft Powerpoint. (TIF 1481 KB) [file 18_2022_4317_MOESM3_ESM.tif]

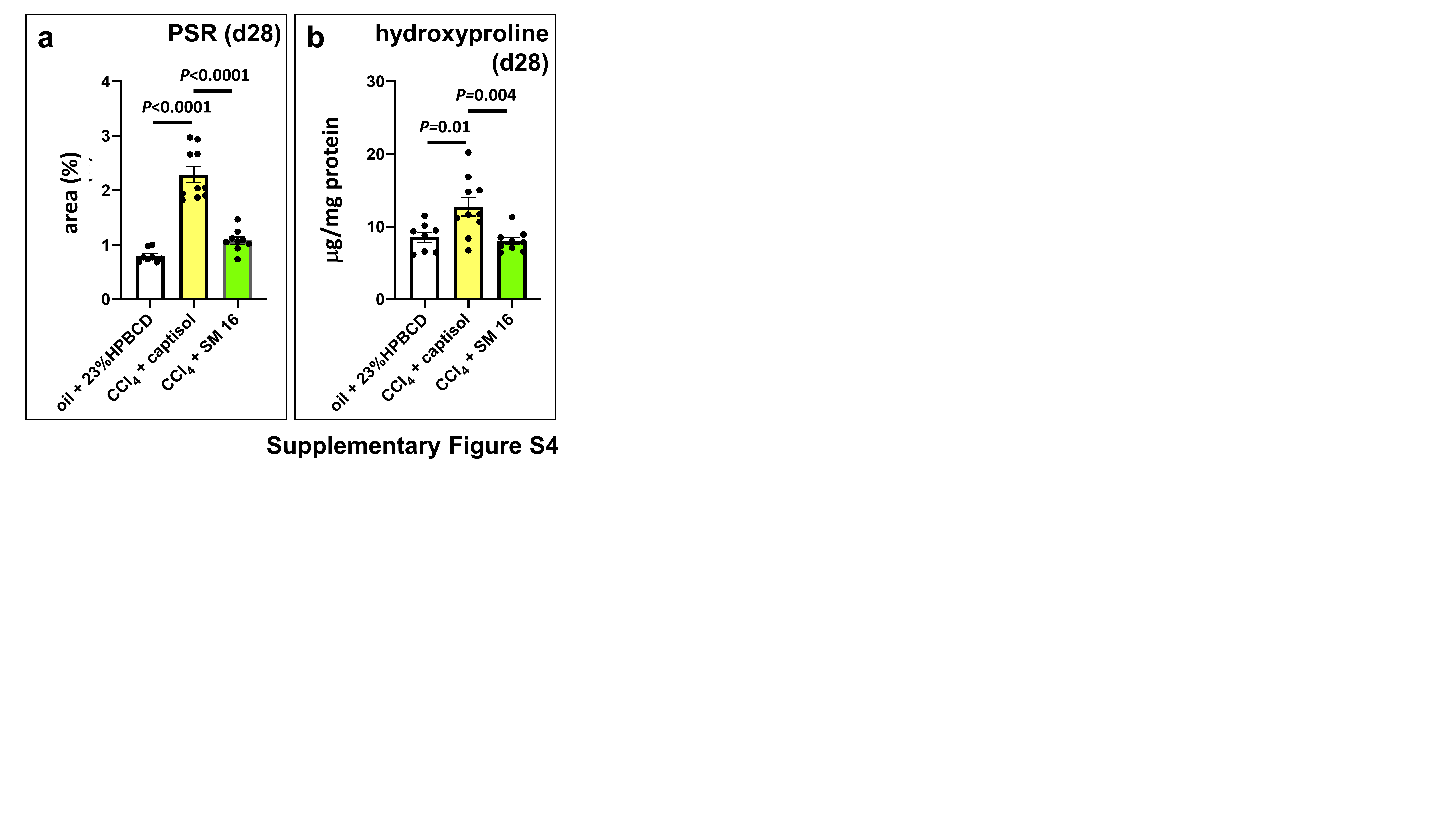

Supplement: Supplementary file 4 — Supplementary file4 Supplementary Fig. S4 Analysis of reference treatment condition in the liver fibrosis model (a,b) Diagrams showing quantification of picro sirius red (PSR) area (a), expressed as a percentage versus tissue area ± sem, and quantification of hydroxyproline content (b) at day (d)28, expressed per weight of protein ± sem in mice treated with mineral oil + 23% HPBCD (white; group 1; n=8), CCl4 + captisol (yellow; group 4; n=10) or CCl4 + SM 16 (green; group 5; n=9). All quantitative data were plotted and analyzed (using Graphpad Prism; version 9.0.1) by one-way ANOVA with Tukey’s post-hoc test. CCl4: carbon tetrachloride; HPBCD: hydroxypropyl-beta-cyclodextrin; captisol: β-Cyclodextrin sulfobutyl ether, sodium salt. Figure composition was made in Microsoft Powerpoint. (TIF 1433 KB) [file 18_2022_4317_MOESM4_ESM.tif]

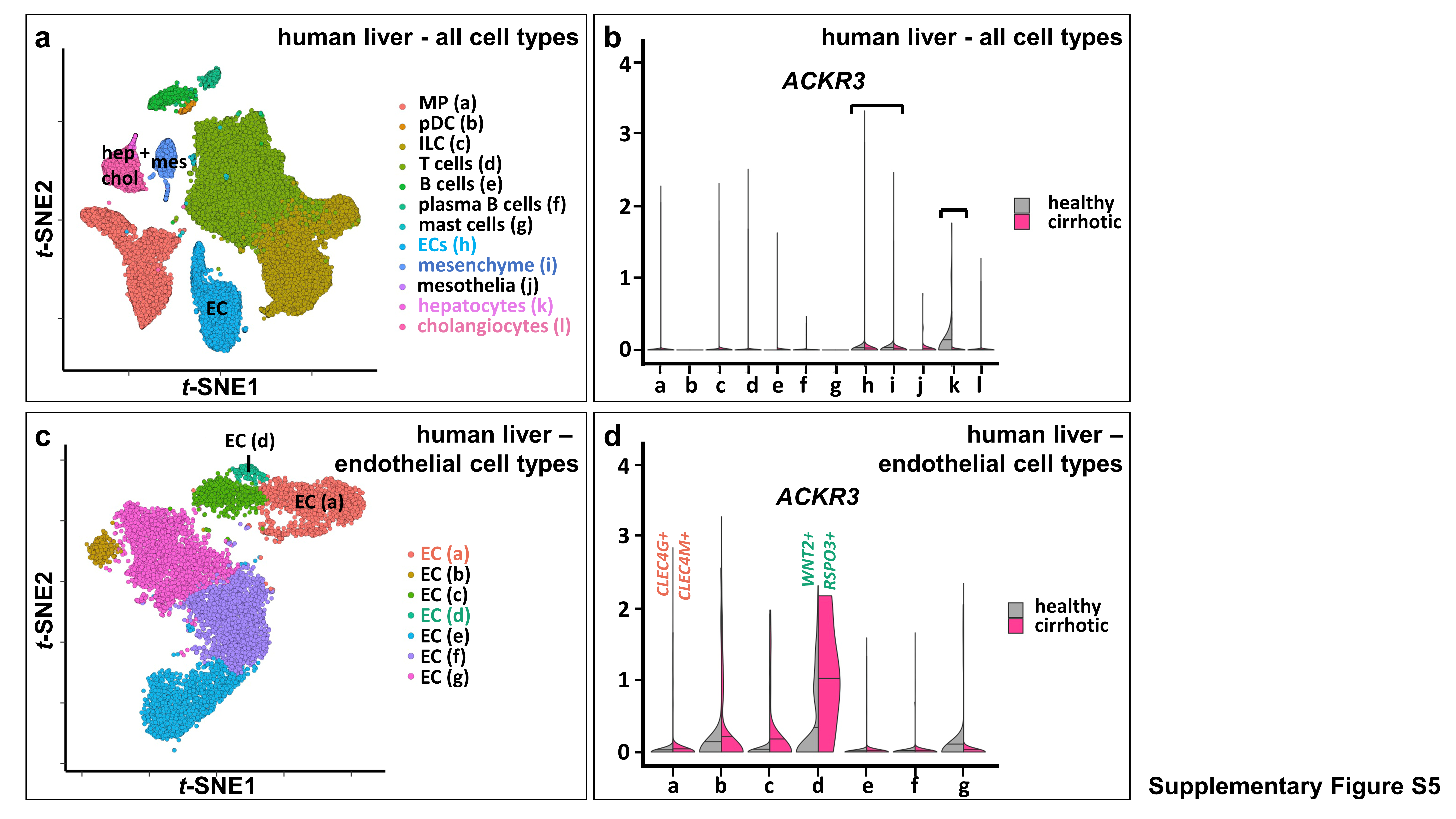

Supplement: Supplementary file 5 — Supplementary file5 Supplementary Fig. S5 Expression of ACKR3 mRNA at single-cell level in healthy and cirrhotic human livers (a,b) Diagrams showing cell cluster analysis by t-Distributed Stochastic Neighbor Embedding (t-SNE; a), revealing 12 cell types represented in human healthy or cirrhotic liver single-cell suspensions and violin plots (b) reporting expression of ACKR3 in each cell cluster in healthy (gray) and cirrhotic livers (purple), revealing expression mostly in the endothelial (EC), mesenchymal (mes) and hepatocyte (hep) clusters. (c,d) Diagrams showing cell cluster analysis by t-SNE (c), revealing 7 EC subtypes represented in human healthy or cirrhotic liver single-cell suspensions and violin plots (d) reporting expression of ACKR3 in each cell cluster in healthy (gray) and cirrhotic livers (purple), revealing expression in most subtypes, including CLEC4G+/CLEC4M+ liver sinusoidal ECs (LSECs; orange) and WNT2+/RSPO3+ ECs from the pericentral area (green). Note the significant increase in the latter cluster in cirrhotic versus healthy livers. MP: mononuclear phagocyte; pDC; plasmacytoid dendritic cell; ILC: innate lymphoid cell; EC: endothelial cell. Data were retrieved from the publicly available gene browser link provided by Ramachandran et al.[31]. Figure composition was made in Microsoft Powerpoint. (TIF 8156 KB) [file 18_2022_4317_MOESM5_ESM.tif]
