# Supplementary material for: Stimulation of the atypical chemokine receptor 3 (ACKR3) by a small-molecule agonist attenuates fibrosis in a preclinical liver but not lung injury model
Source: Cell Mol Life Sci. 2022 May 13;79(6):293. doi: 10.1007/s00018-022-04317-y (PMC9106635; doi:10.1007/s00018-022-04317-y)
Supplement: Supplementary file 6 — Supplementary file6 (DOCX 30 KB) [file 18_2022_4317_MOESM6_ESM.docx]

| **N°** | **Gene** | **NM Probe ID** | **Target sequence** | **Fold change*** | ***P*-value**** |
| --- | --- | --- | --- | --- | --- |
| **Fibrosis-related genes** | | | | | |
| 1 | *Loxl2* | NM_033325.2:3700 | *ttccacttggcgctctggtcttccaaactcccaccacaataacccattcagttctgttctctaaaagcgctctagggcttctggacccaaagttctaagt* | 1.01 | 4.4E-01 |
| 2 | *Golm1* | NM_001035122.2:1940 | *acagaacactatgaagttactgacaatgcgagcacctatggtctgtgtttgtttgattgggcagagtttctagtaagtcactcatctcactaagaaaggc* | 1.46 | 1.1E-03 |
| 3 | *Col4a2* | NM_009932.3:5600 | *ttgtggcaaacctcctgcctcagcttcccagatgctgggattatgggcatgcgccaccacacccagcttggtgcttactcttaacttattacctcagatg* | 0.92 | 1.8E-01 |
| 4 | *Adamts2* | NM_001277305.1:2570 | *tgacatacaaatacatgattcacgaggactccctcaatgttgacgacaacaatgtcctggaagatgatgctgtgcgccacgagtgggccctgaagaagtg* | 1.15 | 2.1E-01 |
| 5 | *Plod2* | NM_001142916.1:2925 | *aacctgaaagcctgggattctgacgcctactggctctatgtaattattaagtcacattgacctctgtgtagggctgttttccaggaattaggaagaaaac* | 1.13 | 2.2E-01 |
| 6 | *Col3a1* | NM_009930.1:4370 | *caagacagtctttgaatatcaaacacgcaaggcaatgagactacccatcatagatatcgcaccctatgacattgggggtcctgatcaagaatttggtgtg* | 1.40 | 6.1E-02 |
| 7 | *Fxyd5* | NM_008761.3:285 | *ccagcagagggcagacaccaaaaaagcccacatccatttttacagcggaccagacttctgcgactactcgtgacaatgtcccagatccagatcaaaccag* | 0.96 | 2.5E-01 |
| 8 | *Arhgap25* | NM_001037727.1:1668 | *gactcaaacgcttcccaaccggaagtgtttcctgacgtccgcattccaaggcaccaccagcagtaaactggaaatctttaaaaatgagttctggtctcca* | 0.87 | 1.5E-01 |
| 9 | *Col1a1* | NM_007742.3:215 | *caatggtgagacgtggaaacccgaggtatgcttgatctgtatctgccacaatggcacggctgtgtgcgatgacgtgcaatgcaatgaagaactggactgt* | 1.49 | 2.2E-02 |
| 10 | *Clec7a* | NM_020008.2:1008 | *ggatgaagaagctgagacttttgtacttgtcatcttcacaaagatggtggcactatcttccagttaggaagtcactagacatggagtgagggcagctcaa* | 1.13 | 2.4E-01 |
| 11 | *Ccr2* | NM_009915.2:2965 | *atgaactaacatagacagctcaggattaacagggacttgtggtttgtggtctgtgggcttatccaagcatggtgatttagactctaaggtccgtctggat* | 0.96 | 3.9E-01 |
| 12 | *Col5a1* | NM_015734.2:6145 | *ccttcagctctcaggataggttcattaaaggtgttaatggaccgttggccgggagtgggggcgggacagtatttgaagatcactttaaaaaaaattcaac* | 0.84 | 1.1E-01 |
| 13 | *Myo1f* | NM_053214.2:3598 | *catgcctggtactgccttcctgtgggttgacttgctatctaagtctccattttctcctgtgtccaatagtagttgctgcaaaacctacctcccagctgcc* | 0.90 | 1.8E-01 |
| 14 | *Thbs2* | NM_011581.2:1250 | *aaatttaaaacagtctgccatcagatcacctgctcacctgcaacttgtgccaacccatcttttgtggaaggcgagtgctgtccatcctgttcacactctg* | 1.03 | 4.3E-01 |
| 15 | *Arl11* | NM_177337.3:878 | *cgtgagcgggcagaacagcttatcctggctatgaacagacggggccagttgaaccttgagaaaaggaagctcagtttaccacacgagaaaccacaattct* | 1.09 | 3.0E-01 |
| 16 | *Lox* | NM_010728.2:2300 | *gatacttgttgctattcgatcccacgctgcttagcttttctgtgggcagaaatgtctaatgtgacaatcagcacatccccattgtgaggtttcacggcat* | 1.24 | 1.5E-01 |
| 17 | *Trem2* | NM_031254.2:7 | *gggcgcctaccctagtcctgactgttgctcaatccaggagcacagttcctgtgggctgagcctgactggcttggtcatctcttttctgcacttcaaggga* | 1.44 | 6.8E-02 |
| 18 | *Eln* | NM_007925.3:3116 | *gtccctgcctcctgttacctaaagctacttcccacatctgggacaccctggagtcagatggctcctcacactgggaatagctcccttgttcttatggaat* | 1.25 | 9.3E-02 |
| 19 | *Tgfb1* | NM_011577.1:1470 | *ggagttgtacggcagtggctgaaccaaggagacggaatacagggctttcgattcagcgctcactgctcttgtgacagcaaagataacaaactccacgtgg* | 0.90 | 1.6E-01 |
| 20 | *Capg* | NM_007599.3:826 | *gaatctgaccaaggtggctgactccagcccttttgcctctgaactgctaattccagatgactgctttgttctggacaacgggctgtgtggcaaaatctac* | 0.85 | 1.8E-01 |
| 21 | *Sdcbp* | NM_001098227.1:158 | *tattctgccaatcccgccagccaagcatttgttctggtggatgcttctgctgctctccctccagatggaaatctgtatcctaaactgtatccagagctct* | 1.08 | 1.4E-01 |
| 22 | *Sla* | NM_001029841.4:634 | *gtgaaacaaagaaaggtttctattcgctgtctgtgagacacaggcaggtgaagcattatcgaatcttccgtcttcccaacaactggtactacatctcacc* | 1.00 | 5.0E-01 |
| 23 | *Timp1* | NM_011593.2:436 | *aagcctctgtggatatgcccacaagtcccagaaccgcagtgaagagtttctcatcacgggccgcctaaggaacggaaatttgcacatcagtgcctgcagc* | 1.09 | 3.1E-01 |
| 24 | *Anxa3* | NM_013470.2:1229 | *cgcctcaaaatctctgcacactgctttcatgcagcactctaaagtgcaagcaaatgcaagacagaacctgtctgcctgataggcattggcatcgttcagt* | 0.85 | 1.9E-01 |
| 25 | *Ly86* | NM_010745.2:725 | *ccagggacagatgttcccagacccaacagatgtaataaaccctcaaaaactatctatttctgaggaccctgagtagtcttgaagccctattgtagtacct* | 1.15 | 1.5E-01 |
| 26 | *Cd68* | NM_001291058.1:780 | *gcacagtggacattcatggcgcagaattcatctcttcgagagctccaagctcccttgggccaaagcttctgctgtggaaatgcaagcatagttctttctc* | 0.90 | 1.6E-01 |
| 27 | *Enc1* | NM_007930.3:4445 | *ttctcaggcttgacacctgtctgaataagagtgattagagccgcataatatccctctcttggctattgaataagtggttcacatacccaaccctgtagaa* | 1.09 | 2.4E-01 |
| 28 | *Nckap1l* | NM_153505.4:424 | *aactttgacttcactcggagctacctggacttaattgtgacgtacacctcagtcattttactcctgtcacggattgaggaccgcaggatactcattggca* | 0.97 | 4.3E-01 |
| 29 | *Arpc1b* | NM_023142.2:1291 | *taagggctgctttgctaaatgtttctaggatgcagtacactgctacaaaggaagaggcaggaggggaactgcctatttaacaaaatgtgccttttaaaga* | 1.04 | 3.4E-01 |
| 30 | *Gdf15* | NM_011819.2:715 | *gctgagcatgtgcgtgggcgagtgtccccacctgtatcgctccgcgaacacgcatgcgcagatcaaagcacgcctgcatggcctgcagcctgacaaggtg* | 1.12 | 1.9E-01 |
| 31 | *Laptm5* | NM_010686.3:36 | *ccggtcagacagacatgctgttgtttcaacatccgagtcgccaccatagccctggccatttaccacatagtcatgagtgtcctgctgttcattgagcatg* | 0.90 | 2.0E-01 |
| 32 | *Lgals3* | NM_001145953.1:1005 | *catccatttaataaagtctcatgctgagagatacccatcgctttgggggtttttatgatactggatgtcaaatcttaggactgctcgtgactgctaggca* | 0.58 | 9.1E-03 |
| 33 | *Unc93b1* | NM_019449.2:1060 | *gcccttcaaacacgtgcgtgactttcgcttacgccatctggtgcccttctttatctacagtggctttgaggtgctctttgcctgcactggttttgccctg* | 0.81 | 2.5E-02 |
| 34 | *Tnc* | NM_011607.1:5665 | *gcaaaaatggacgtgaggacttctatcgcaactggaaggcctatgctgctgggtttggagaccgcagagaagaattttggcttggactggataacctgag* | 0.84 | 2.9E-01 |
| 35 | *Cxcl16* | NM_023158.6:679 | *cgcagggtactttggatcacatccgaaaatacctgaaagcatttcatcgttgtccattctttatcaggttccagttgcagtccaaaagcgtgtgtggggg* | 1.03 | 4.1E-01 |
| 36 | *Axl* | NM_009465.3:3820 | *gagattctaaaggtccacagtctagagtattaggtacgactccaagggtgggcgcttgtagccatcctaagtcctttccctccttaagcacctatgctcc* | 0.96 | 3.5E-01 |
| 37 | *Clec4n**** | NM_001190320.1:10 | *ctctgacttctgggtttgcagcattggcccgctctgtggcatttaactcaagtgtgtgtggaagttgattctgaactctggcctctttgacagaagccag* | 0.77 | 4.9E-02 |
| 38 | *Lilrb4* | NM_013532.3:348 | *accaggaataaggccaagttcaacattccaagcatgacaacctcatatgcaggcatatataagtgttactatgagagtgctgctggcttctcagagcaca* | 0.94 | 3.3E-01 |
| 39 | *Ms4a6a* | NM_027209.3:583 | *agagcaaggagcttagaccaactgaatatcattactaccaattcttggacaggaacgagtgctttgccgccaagtctgttctggctggagtcttttcact* | 1.20 | 9.6E-02 |
| 40 | *Ccr5* | NM_009917.5:1340 | *ggagcagggagaacgagtcttttatcagggccgggaaatatgcacaaagagacttgaggcaggtgccatgacccatatgcaaagggacggacacagggcc* | 0.86 | 1.6E-01 |
| 41 | *Pld4* | NM_178911.4:225 | *ctagctgtactgggactcagctctgtgactctcatcttgttcctgtggcaaggggccacttctttcaccagtcatcggatgttccctgaggaagtgccct* | 0.93 | 3.0E-01 |
| 42 | *C1qb* | NM_009777.2:865 | *gtgccaacagcatcttcactggctttctgcttttccctgacatggatgcgtaatcacggggtcaaattacacctatccaacaccatcttcctgcctccct* | 0.92 | 2.5E-01 |
| 43 | *Il1rn* | NM_031167.5:224 | *caaccagctcattgctgggtacttacaaggaccaaatatcaaactagaagaaaagatagacatggtgcctattgaccttcatagtgtgttcttgggcatc* | 0.76 | 1.5E-01 |
| 44 | *Slc11a1* | NM_013612.2:945 | *catgtacttcctgattgaggccaccatcgccctatcggtgtccttcatcatcaacctcttcgtcatggctgtttttggtcaggccttctaccagcaaacc* | 0.88 | 1.4E-01 |
| 45 | *Rassf4* | NM_178045.4:4845 | *gtgtaacttatggattcacaggcttccttccgtggcctgagaagcaacaaggcagaagatcctcatatctcaagtaggagtaagacgtttctacatcact* | 0.93 | 3.1E-01 |
| 46 | *Serpine1* | NM_008871.2:1822 | *aggggcaacggatagacagatcaaatggtggcccaatagcgagccttctccctgctccctcccttgacacagcttgcttatgttatttcagagtgtaggt* | 0.22 | 8.2E-02 |
| 47 | *Mmp9* | NM_013599.2:1570 | *cctctacagagtctttgagtccggcagacaatccttgcaatgtggatgtttttgatgctattgctgagatccagggcgctctgcatttcttcaaggacgg* | 1.13 | 2.5E-01 |
| 48 | *Slc15a3* | NM_023044.2:1078 | *ggttctattggagcatcaacctgggtgccatattgtccctgttggtggtggctttcatcgagcagaacatcagcttcctatggggctacagcatcatcgt* | 0.81 | 3.0E-02 |
| 49 | *Sec24d* | NM_027135.2:2865 | *tcttatggtttggagtgggcagcccaccagagctgattcagggaatatttaatgtgccatcgtttgcacatatcaacacagatatgacatcgctgcctga* | 1.22 | 2.7E-02 |
| 50 | *Cd86* | NM_019388.3:251 | *caaaacataagcctgagtgagctggtagtattttggcaggaccagcaaaagttggttctgtacgagcactatttgggcacagagaaacttgatagtgtga* | 0.88 | 1.3E-01 |
| 51 | *Ccr9* | NM_009913.6:820 | *tataccatcatcattcataccttggtacaggccaagaagtcatccaagcacaaggccctcaaggtgaccatcactgtcctcactgtcttcattatgtctc* | 1.04 | 4.1E-01 |
| 52 | *Cxcl10* | NM_021274.1:115 | *aggacggtccgctgcaactgcatccatatcgatgacgggccagtgagaatgagggccatagggaagcttgaaatcatccctgcgagcctatcctgcccac* | 0.74 | 3.7E-02 |
| **Housekeeping genes** | | | | | |
| 1 | *Actb* | NM_007393.3:71 | *agttcgccatggatgacgatatcgctgcgctggtcgtcgacaacggctccggcatgtgcaaagccggcttcgcgggcgacgatgctccccgggctgtatt* | - | - |
| 2 | *Hprt1* | NM_013556.2:30 | *tgctgaggcggcgagggagagcgttgggcttacctcactgctttccggagcggtagcacctcctccgccggcttcctcctcagaccgctttttgccgcga* | - | - |
| 3 | *Ldh* | NM_010699.2:1354 | *ctgcaggcttcgattacccctgtgagcctgctgcattgctgccctgcaccaaacatgcctaggccgacgagttcccagttaagtcgtataacctggctcc* | - | - |
| 4 | *Tbp* | NM_013684.3:70 | *gtggcgggtatctgctggcggtttggctaggtttctgcggtcgcgtcattttctccgcagtgcccagcatcactatttcatggtgtgtgaagataaccca* | - | - |

**Supplementary Table 1. nCounter probe list and numeric data.** Significantly altered genes are shaded in green. *: CCl_4_ + compound **18a** *vs.* CCl_4_ + 23% HPBCD; **corrected *P*-value; ****Clec4n* is the mouse orthologue of human *CLEC6A.*
