# Supplementary material for: Stimulation of the atypical chemokine receptor 3 (ACKR3) by a small-molecule agonist attenuates fibrosis in a preclinical liver but not lung injury model
Source: Cell Mol Life Sci. 2022 May 13;79(6):293. doi: 10.1007/s00018-022-04317-y (PMC9106635; doi:10.1007/s00018-022-04317-y)
Supplement: Supplementary file 7 — Supplementary file7 (DOCX 14 KB) [file 18_2022_4317_MOESM7_ESM.docx]

**Supplementary Table 2. Pharmacokinetic profile of compound** 18a **after subcutaneous administration of 30mg/kg**

| **compound** | **T_max_ [h]** | **t_1/2_ [h]** | **C_max_ [ng/mL]** | **AUC [ng/mL·h]** |
| --- | --- | --- | --- | --- |
| 18a | 0.25 | 5.2 | 6,116 ± 1570.98 | 6,699 |

Pharmacokinetic parameters were calculated from plasma concentration-time data in male C57BL/6 mice (*n*=3). C_max_ is reported as the mean ± SD.
